# Effectiveness of Mobile Medical Apps in Ensuring Medication Safety Among Patients With Chronic Diseases: Systematic Review and Meta-analysis

**DOI:** 10.2196/39819

**Published:** 2022-11-22

**Authors:** Ting ting Zhou, Rui Wang, Si jia Gu, Li ling Xie, Qing hua Zhao, Ming zhao Xiao, Yu lu Chen

**Affiliations:** 1 Department of Nursing The First Affiliated Hospital of Chongqing Medical University Chongqing China; 2 Hepatobiliary Surgery The Second Affliated Hospital of Chongqing Medical University Chongqing China

**Keywords:** mobile application, medication safety, systematic review, meta-analysis, mobile health, mHealth, health app, adherence, pharmaceutical, drug safety, medication error, drug error, review methodology, search strategy, eHealth, digital health, adverse event, adverse effect

## Abstract

**Background:**

Along with the rapid growth of the global aging society, the mobile and health digital market has expanded greatly. Countless mobile medical apps (mmApps) have sprung up in the internet market, aiming to help patients with chronic diseases achieve medication safety.

**Objective:**

Based on the medication safety action plans proposed by the World Health Organization, we aimed to explore the effectiveness of mmApps in ensuring the medication safety of patients with chronic diseases, including whether mmApps can improve the willingness to report adverse drug events (ADEs), improve patients' medication adherence, and reduce medication errors. We hoped to verify our hypothesis through a systematic review and meta-analysis.

**Methods:**

The meta-analysis was performed in strict accordance with the PRISMA (Preferred Reporting Items for Systematic Reviews and Meta-Analyses) guidelines and included literature searched from 7 databases—PubMed, Web Of Science, Embase, CINAHL, China National Knowledge Infrastructure, Wanfang, and SinoMed. The publication time was limited to the time of database establishment to April 30, 2022. Studies were screened based on inclusion and exclusion criteria. The data extracted included authors, years of publication, countries or regions, participants’ characteristics, intervention groups, and control groups, among others. Our quality assessment followed the guidelines of the *Cochrane Handbook for Systematic Reviews of Interventions, Version 6.3*. RevMan 5.2 software (Cochrane Collaboration) was used to analyze the statistical data, and a sensitivity analysis was performed to assess data stability. The degree of stability was calculated by using a different statistical method and excluding large-sample studies from the analysis.

**Results:**

We included 8 studies from 5 countries (China, the United States, France, Canada, and Spain) that were published from January 1, 2014, to December 31, 2021. The total number of participants was 1355, and we analyzed the characteristics of included studies, each app’s features, the risk of bias, and quality. The results showed that mmApps could increase ADE reporting willingness (relative risk [RR] 2.59, 95% CI 1.26-5.30; *P*=.009) and significantly improve medication adherence (RR 1.17, 95% CI 1.04-1.31; *P*=.007), but they had little effect on reducing medication errors (RR 1.54, 95% CI 0.33-7.29; *P*=.58)*.*

**Conclusions:**

We analyzed the following three merits of mmApps, with regard to facilitating the willingness to report ADEs: mmApps facilitate more communication between patients and physicians, patients attach more importance to ADE reporting, and the processing of results is transparent. The use of mmApps improved medication adherence among patients with chronic diseases by conveying medical solutions, providing educational support, tracking medications, and allowing for remote consultations. Finally, we found 3 potential reasons for why our medication error results differed from those of other studies.

**Trial Registration:**

PROSPERO International Prospective Register of Systematic Reviews CRD42022322072; https://www.crd.york.ac.uk/prospero/display_record.php?RecordID=322072

## Introduction

Medication safety has been a major concern of international organizations and government agencies. The World Health Organization (WHO) [1] selected medication safety as the theme for World Patient Safety Day 2022. *Medication safety* refers to ensuring that the right medications are used by the right patients in the right way. This topic was mentioned again after the WHO launched the “Third Global Patient Safety Challenge: Medication Without Harm” in 2017 [2], reflecting the extremely important role of medication safety in ensuring patient safety. According to the WHO [3], more than 60% of patients with chronic diseases in the world have long-term disease states and take multiple drugs. As such, medication safety has become a significant issue. If adverse drug events (ADEs) continue to occur, they will result in more than US $420 million in economic losses; increase disease burden and rehospitalization rates; and result in a series of adverse consequences, such as disability, fainting, and even death [4,5]. Studies have shown that approximately 80% of medication errors are preventable [3]. In order to ensure medication safety, the WHO proposed the following specific action plans [6]: (1) engaging patients and families in reporting ADEs, (2) improving patients’ medication adherence, and (3) reducing medication errors. Therefore, this study was guided by the WHO’s medication safety action plans and explored how to ensure patient safety in terms of the above three action plans.

In recent years, along with the rapid growth of the global aging society, the mobile and health digital market has greatly expanded. Countless mobile medical apps (mmApps) have sprung up in the internet market, aiming to help patients with chronic diseases achieve medication safety. By the end of 2020, around 3.25 million mmApps were downloaded from common app stores (ie, the Android and Apple app stores)—a full 50% increase from 10 years ago [7]. These mmApps were invented and created with the help of big data and 5G technology, and they have functions such as medication reminders, self-diagnosis functions, ADE reporting functions, information acquisition and consultation functions, and wellness management functions [8]. However, most scholars mainly focus on the role of mmApps in improving medication adherence [9-11], thus ignoring their equally important role in ADE reporting and medication error reduction.

We hypothesized that mmApps could effectively guarantee medication safety by facilitating the reporting of ADEs, improving medication adherence, and reducing medication errors, and we validated our hypothesis through a systematic review and meta-analysis.

## Methods

### Study Protocol and Registration

The systematic review and meta-analysis were conducted by following the PRISMA (Preferred Reporting Items for Systematic Reviews and Meta-Analyses) guidelines [12], and our study was registered in PROSPERO (an international database of prospectively registered systematic reviews; registration number: CRD42022322072) on April 2022.

### Search Strategy

We conducted a systematic search of PubMed, Web Of Science, Embase, CINAHL, China National Knowledge Infrastructure, Wanfang (a traditional Chinese literature database), and SinoMed (a Chinese biomedical database). The publication time was limited to the time of database establishment to April 30, 2022. The publication language was restricted to Chinese and English. We conducted the search by using a combination of keywords and MeSH (Medical Subject Headings) terms. For example, our search strategy for PubMed involved using the following search string: “(((medication errors OR look-alike sound-alike medication errors OR high-alert drug error OR drug use error*) OR (medication adherence OR drug adherence OR medication persistence OR medication compliance OR drug compliance)) OR (adverse drug event* OR ADE OR drug related side effects AND adverse reaction* OR drug side effects OR adverse drug reaction* OR side effects of drugs OR drug toxicity)) AND (mobile application* OR mobile App* OR portable software App*OR smartphone Apps OR portable electronic Apps OR portable electronic application).” More research details are provided in Multimedia Appendix 1.

### Inclusion and Exclusion Criteria

The inclusion criteria were as follows: (1) age≥18 years, (2) at least one chronic disease, (3) an intervention group that used mmApps and a control group that underwent usual care (ie, without using mmApps), and (4) clinical trials.

The exclusion criteria were as follows: (1) the presence of confounding factors in randomized controlled trials, such as mixed methods (a combination of qualitative and quantitative methods); (2) the experimental procedures are not clear and transparent (eg, the intervention procedures or results are not clearly expressed); and (3) duplicate publications and publications for which we were unable to contact the authors.

### Data Extraction

We created a Microsoft Excel spreadsheet to extract key information from the included studies, including authors, years of publication, countries or regions, participants’ characteristics, intervention groups, control groups, names of apps, functions of apps, intervention durations, outcomes, and measurement tools.

### Participants

The studies involved participants (aged ≥18 years) with a chronic disease, including hypertension, diabetes, coronary heart disease, arthritis, and other single chronic diseases. However, studies that involved patients with multiple coexisting chronic diseases were also included.

### Intervention

Owing to different intervention measures, participants were divided into control groups and intervention groups. Control groups underwent usual care, such as keeping a medication diary, attending regular follow-up visits, and attending medication safety lectures. Intervention groups used mmApps in addition to undergoing the usual care provided to control groups. These mmApps included, but were not limited to, smartphone mmApps, iPad (Apple Inc) mmApps, and WeChat (Tencent Holdings Limited) mini-programs.

### Outcome Measures

In terms of medication safety outcomes, we focused on medication adherence, which was calculated by examining medication doses and frequencies in patients with chronic diseases to verify whether they matched physicians’ prescriptions. Another important outcome was the rate of ADE reporting, which depended on whether patients reported the occurrence of ADEs. The third outcome was medication errors. Despite using mmApps, there was still the possibility of medication errors; taking the wrong pill or the wrong dose, giving medications to the wrong patient, and taking medications in the wrong manner or at the wrong time were considered medication errors.

### Quality Assessment

The literature quality assessment was based on 7 criteria from the *Cochrane Handbook for Systematic Reviews of Interventions, Version 6.3*, which was updated by the Cochrane Collaboration in 2022 [13]. The reviewers made a separate judgment for each item (ie, low risk of bias, high risk of bias, or unclear risk of bias). If a study fully met these criteria, the likelihood of various biases was low, and the quality grade was “A.” If these criteria were partially met, the probability of bias was moderate, and the quality grade was “B.” If these criteria were not met at all, the probability of bias was high, and the quality grade was “C.” Two investigators with evidence-based training were invited to simultaneously evaluate the quality of the included literature, and a third investigator was consulted when disagreements occurred. Articles with an overall quality level of A or B were included, and articles with an overall quality level of C were excluded.

### Statistical Analysis

RevMan 5.2 software (Cochrane Collaboration) was used to analyze the data. Weighted mean differences were used to analyze the effect sizes of continuous variables, and relative risk (RR) was used to analyze the effect sizes of dichotomous variables. Further, 95% CIs were used to represent the sizes of combined effects. Additionally, heterogeneity was tested. If *I*^2^ was <50%, the homogeneity was considered to be good, and a fixed effect model was used for the analysis. If *I*^2^ was >50%, the heterogeneity was considered to be large, and a random effect model was used for the analysis. Heterogeneity was explained in terms of clinical and methodological heterogeneity, and a subgroup analysis was performed, if necessary.

### Sensitivity Analysis

A sensitivity analysis was performed to assess data stability. The degree of stability was calculated by using a different statistical method and excluding large-sample studies from the analysis, so as to verify whether our results were robust and reliable.

## Results

### Identified and Included Studies

**Figure 1:**
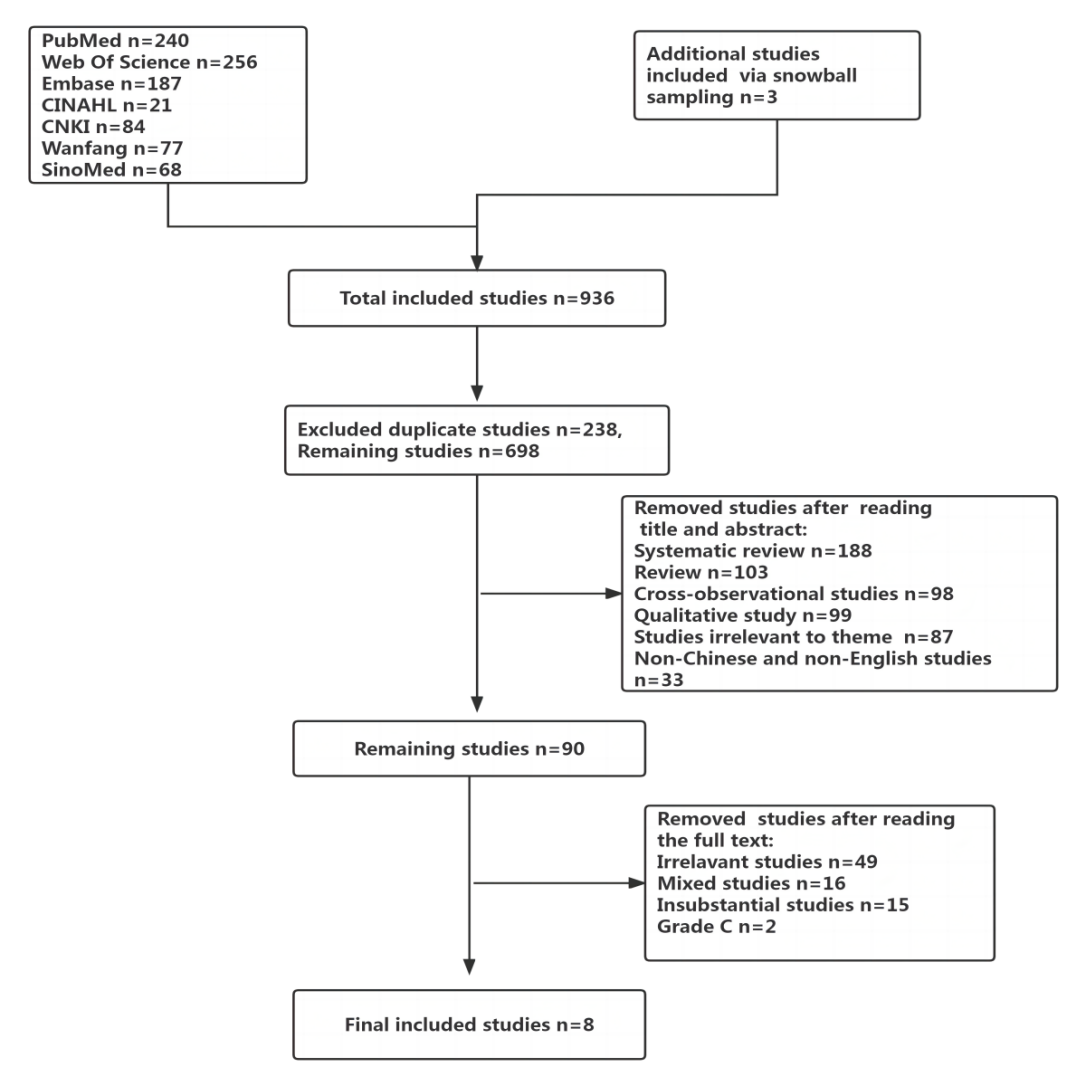
Flow diagram of the study retrieval and selection process.

We searched 7 databases and obtained 936 articles. The first step was to exclude duplicate articles via NoteExpress software (AegeanSoftware Corp), which left us with 698 articles. In the second step, studies were excluded by screening titles and abstracts, which left us with 90 articles. In the third step, studies were excluded by carefully screening the full text of articles. Finally, we included 8 articles. The retrieval and selection process is shown in Figure 1.

### Characteristics of Included Studies

There were 1355 participants in the 8 studies, which were from 5 countries (China, the United States, France, Canada, and Spain). The publication times ranged from January 1, 2014, to December 31, 2021. The median sample size was 169 (range 61-268), and the following chronic diseases were included in the studies: pulmonary tuberculosis [14], renal cancer or prostate cancer [15], influenza [16], multiple sclerosis [17], oral cancer [18], and multiple chronic diseases [19-21]. The median intervention duration was 6 (range 1-25) months. The intervention groups used mmApps, and the control groups underwent usual care (ie, they did not use mmApps). As for the outcomes, 3 studies reported ADEs [16,17,19], 4 studies measured medication adherence [14,15,18,20], and 4 studies calculated the frequency of medication errors [15,17,20,21]. Further details are shown in Table 1.

**Table 1:** Detailed information of included studies.

Authors and year	Country	Participants	Sample size	Names of apps	App functions	Intervention vs control	Duration	Outcomes
Mira et al [20], 2014	Spain	Older patients taking multiple medications	N=99 (CG^a^: n=48; IG^b^: n=51)	ALICE	Providing prescriptions and medical advice, showing medication images, and sending multiple reminders	mmApp^c^ vs UC^d^	3 months	Medication errors (CG: n=5, 10.4%; IG: n=8, 15.7%) and medication adherence (MMAS-4^e^)
Mira et al [21], 2015	Spain	Older patients with multiple chronic diseases	N=61 (CG: n=30; IG: n=31)	TUMEDICIN	Providing information on the purpose of a given medicine, daily doses, possible adverse effects, and main cautions	mmApp vs UC	3 months	Medication errors (CG: n=13, 43.3%; IG: n=6, 19.4%)
Wei et al [14], 2019	China	Patients with pulmonary tuberculosis	N=300 (CG: n=l40; IG: n=160)	E-monitor Box and WeChat (Tencent Holdings Limited)	Monitoring patients’ adherence history and outpatient visits, reporting ADEs^f^, and reminding patients to take their medications	mmApp vs UC	6 months	High medication adherence (CG: n=101, 72.1%; IG: n=96, 60%)
Agboola et al [15], 2014	United States	Patients with renal cancer or prostate cancer	N=150 (CG: n=76; IG: n=74)	CORA^g^	Providing coaching for self-efficacy in self-care and reporting and managing symptoms	mmApp vs UC	3 months	Medication errors (CG: n=23, 30.3%; IG: n=14, 18.9%) and medication adherence (MMAS-8^h^)
Wilson et al [16], 2016	Canada	Patients reporting adverse events after influenza vaccination	N=152 (CG: n=76; IG: n=76)	CANVAS	Reporting ADEs spontaneously and evaluating user experience	mmApp vs UC	1 month	ADE reporting cases (CG: n=15, 19.7%; IG: n=35, 46.1%)
Montastruc et al [19], 2018	France	Patients with chronic diseases	N=268 (CG: n=133; IG: n=135)	VigiBIP	Providing spontaneous reports of pharmacy vigilance and drug safety information	mmApp vs UC	25 months	ADE reporting cases (CG: n=59, 44.4%; IG: n=94, 69.6%)
Defer et al [17], 2021	France	Patients with multiple sclerosis	N=159 (CG: n=68; IG: n=91)	My eReport	Not mentioned	mmApp vs UC	6 months	ADE reporting cases (CG: n=5, 7.4%; IG: n=43, 47.3%) and medication error cases (CG: n=3, 0.4%; IG: n=64, 70.3%)
Greer et al [18], 2020	United States	Patients undergoing oral cancer therapy	N=166 (CG: n=86; IG: n=80)	Smartphone mobile app	Medication plans with reminders, a symptom reporting module, and patient education	mmApp vs UC	3 months	High medication adherence cases (CG: n=66, 76.7%; IG: n=69, 86.3%)

^a^CG: control group.

^b^IG: intervention group.

^c^mmApp: mobile medical app.

^d^UC: usual care (ie, did not use a mobile medical app).

^e^MMAS-4: Morisky Medication Adherence Scale-4 item.

^f^ADE: adverse drug event.

^g^CORA: Chemotherapy Assistant.

^h^MMAS-8: Morisky Medication Adherence Scale-8 item.

### Features of Apps in Included Studies

In all 8 studies, the intervention groups used mmApps, and each app had its own characteristics. Mira et al [20,21] focused on the impact of mmApps on medication self-management among patients with chronic diseases. They invented an app called “ALICE” in 2014, which could provide prescriptions and medical advice, display medication images, and send multiple reminders. In 2015, the mmApp was improved and optimized by adding QR code scanning functions and renamed as “TUMEDICIN.” The app could scan QR codes on medical products and provide information, including information on the purpose of a medication, daily doses, possible adverse effects, and main precautions.

Wei et al [14] implemented an electronic monitoring intervention based on WeChat (a local social software that has achieved large-scale population coverage) that could be used to monitor patient adherence and outpatient clinic visits, report adverse drug reactions, and remind people to take their medicine. US scholars mainly concentrated on medication safety for patients undergoing cancer treatment. Agboola et al [15] developed Chemotherapy Assistant (CORA) to help patients with renal cancer or prostate cancer report and manage their symptoms by improving their self-efficacy. Greer et al [18] used a smartphone mobile app to execute medication plans, and the app included reminders, a symptom reporting function, and patient education.

Wilson et al [16] and Montastruc et al [19] showed great interest in the reporting of ADEs and independently developed CANVAS (an app for automatically reporting ADEs and evaluating user experience) and VigiBIP (an app for spontaneously reporting pharmacy vigilance and drug safety information), respectively. All of the mmApps reported in this study were developed based on real-time communication technology and intelligent automatic identification technology, which simplifies complex clinical practices and provides convenience for medical staff.

### Meta-analysis of Intervention Efficacy

#### mmApps Facilitated ADE Reporting

In the Defer et al [17] study, participants reported adverse drug reactions with My eReport, through which they submitted their basic information (name, age, weight, gender, and medical history), medication information (drug name, method, dose, and date of taking medication), and adverse reactions (descriptions of the start of the reaction, processes for managing the reaction, outcomes of the reaction, and information on how users felt after the reaction). Montastruc et al [19] studied spontaneous adverse drug reaction reports that were received through VigiBIP, a free smartphone app for reporting adverse drug reactions and requesting drug safety information. Wilson et al [16] provided a mechanism for automatically reporting ADEs. All of their findings were recorded on a private cloud server. Paying more attention to pharmacovigilance can help prevent ADEs from happening again.

**Figure 2:**
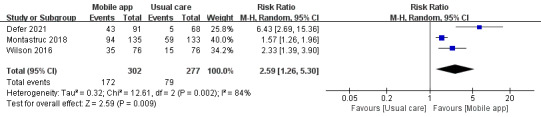
Forest plot of the effects of mobile apps and usual care on adverse drug event reporting [16,17,19]. M-H: Mantel-Haenszel.

In total, 3 studies compared the effects of mmApps and usual care on the reporting of ADEs. A total of 579 patients were included. These studies had large heterogeneity, and we used a random effect model to analyze their results. Our results showed that mmApps had a statistically significant effect on ADE reporting (RR 2.59, 95% CI 1.26-5.30; *P*=.009; Figure 2).

#### mmApps Improved Medication Adherence

There were 4 studies in which the outcome was medication adherence, but only 2 of these studies were included in the meta-analysis. The other two studies were excluded due to the large heterogeneity in the measurement tools used for assessing medication adherence (one study tool was the Morisky Medication Adherence Scale [MMAS]-4 item [20], and the other was the MMAS-8 item [15]). So far, the most popular measurement tool for assessing medication adherence is the MMAS [22], which is a self-reported medication adherence scale that was first proposed by Morisky et al [22] in 1986. After more than 20 years of development, it has been revised from the original 4-item scale to the 8-item scale that most people use now. However, the MMAS is susceptible to the influence of patients' memory bias; with the increase of age, memory declines, and the reliability of the MMAS decreases. Further, different measurement tools may have different impacts on the results of medication adherence. As such, we decided to include the Greer et al [18] and Wei et al [14] studies, as they assessed the same outcome and used the same measurement tools.

**Figure 3:**
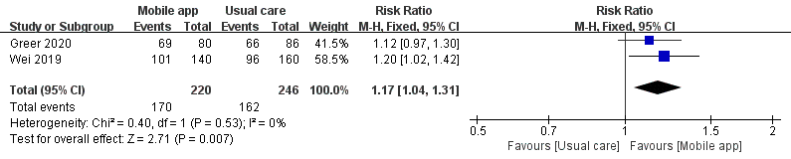
Forest plot of the effects of mobile apps and usual care on medication adherence [14,18]. M-H: Mantel-Haenszel.

A total of 2 studies compared the effects of mmApps and usual care on medication adherence. A total of 466 patients were included. The heterogeneity was small, and we used a fixed effect model to analyze their results. Our results showed that mmApps had a statistically significant effect on medication adherence (RR 1.17, 95% CI 1.04-1.31; *P*=.007; Figure 3).

#### mmApps Scarcely Prevented Medication Errors

Agboola et al [15] and Defer et al [17] developed CORA and My eReport, respectively, which were personalized, mobile phone–based self-management apps for helping patients with renal cancer or prostate cancer on oral anticancer medications. These apps could reduce medication errors though health education (ie, they increased patients’ understanding of medications, drug side effects, safe storage, best practices, and home security for antitumor drugs), psychological support, the early reporting of symptoms, and disease management. Mira et al [20,21] ensured and promoted safer medications for older patients via the use of QR and European Article Number-13 codes. Their results showed that 13 of the 30 (43%) patients in the control group experienced at least one medication error within 1 year, while 6 of the 31 (19%) patients in the intervention group experienced medication errors. Of the 6 medication error cases, 2 were the result of confusion related to incorrect medications, 1 was related to side effects resulting from drug mixing, 2 were related to taking medications at incorrect times, and 1 was related to taking a higher than prescribed medication dose.

**Figure 4:**
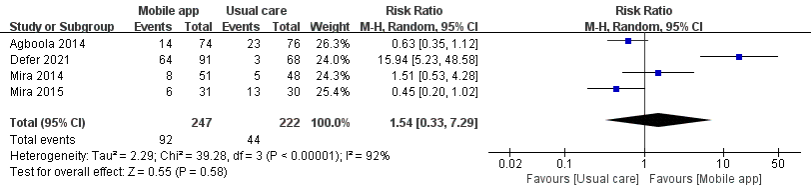
Forest plot of the effects of mobile apps and usual care on medication errors [15,17,20,21]. M-H: Mantel-Haenszel.

In total, 4 studies compared the effects of mmApps and usual care on medication errors. A total of 469 patients were included. These studies had large heterogeneity, and we used a random effect model to analyze their results. Our results showed that mmApps did not have a statistically significant effect on medication errors (RR 1.54, 95% CI 0.33-7.29; *P*=.58; Figure 4).

### Risk of Bias and Quality Assessment

**Figure 5:**
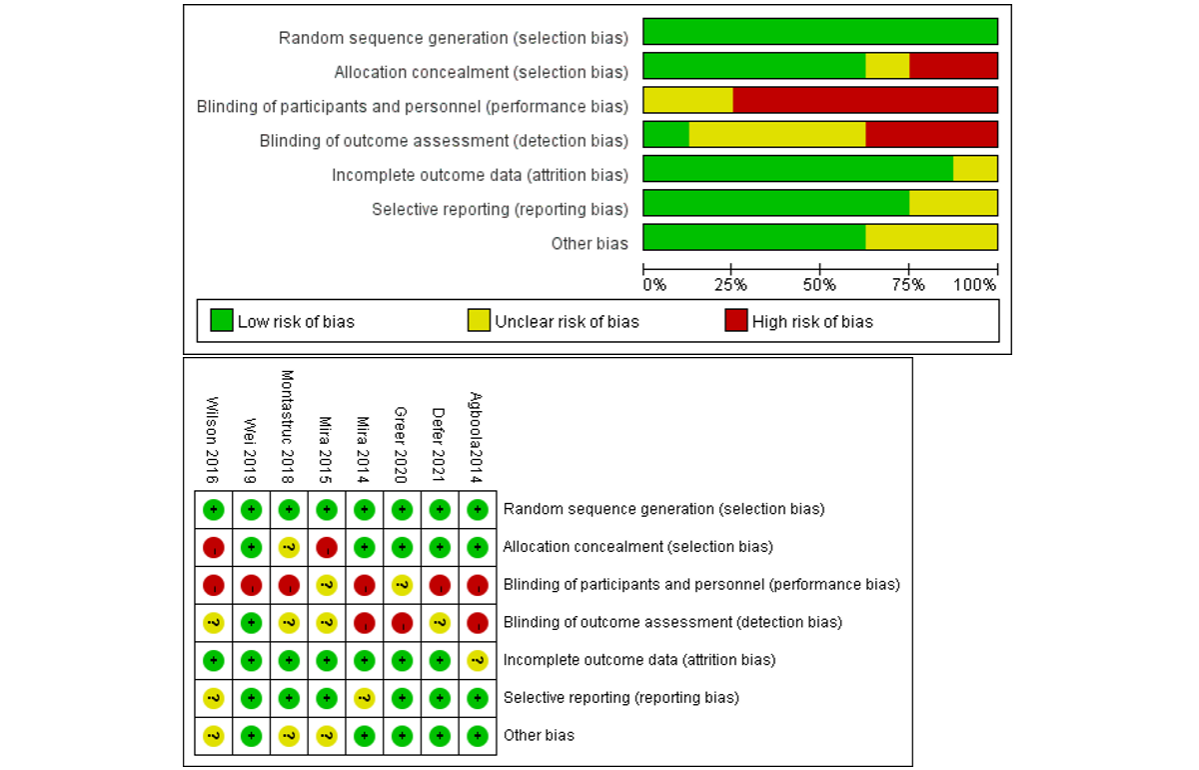
Risk of bias summary for each included study [14-21].

All 8 articles reported adequate random sequence generation and thus had a low risk of bias in this regard. Further, 5 studies reported allocation concealment, so the risk of bias was low in this regard, and 2 studies did not mention concealment and were rated as having a high risk of bias in this regard. As for performance bias, 6 studies were not blinded, so they were rated as having a high risk of bias, and the presence of blinding in the other two studies was unclear. Most of the studies (7/8, 88%) reported results and follow-ups, so the risk of attrition bias was low. With regard to reporting bias, 2 studies had an unclear risk of bias because they did not clearly express participant characteristics, and the other studies had a low risk of bias. In summary, all 8 studies partially met the quality criteria, and their quality grade was “B.” Therefore, they were all included in this study. The specific risk of bias and quality evaluation results are shown in Figure 5.

### Sensitivity Analysis

To explore the stability and the degree of stability of our study’s results, we used a different statistical method to analyze the results. It was found that after changing the statistical method for analyzing different outcome indicators, there were no differences in ADE reporting and medication adherence results, indicating low sensitivity and robust and reliable results, as shown in Table 2.

As for medication error results, our original results (RR 1.54, 95% CI 0.33-7.29; *P*=.58) changed when we removed large-sample studies (RR 0.69, 95% CI 0.38-1.24; *P*=.21) [17]. The results did not change substantially, indicating low sensitivity and robust and reliable results.

**Table 2:** Sensitivity analysis of different outcome indicators.

Models			Relative risk (95% CI)		*Z* value		*P* value
**Adverse drug event reporting**							
	Random effect model	2.59 (1.26-5.30)		2.59		.009	
	Fixed effect model	2.06 (1.67-2.53)		6.83		＜.001	
**Medication adherence**							
	Random effect model	1.16 (1.04-1.29)		2.65		.008	
	Fixed effect model	1.17 (1.04-1.31)		2.71		.007	
**Medication errors**							
	Model that retained large-sample studies	1.54 (0.33-7.29)		0.55		.58	
	Model that did not retain large-sample studies	0.69 (0.38-1.24)		1.25		.21	

## Discussion

### Principal Findings

The results of our study verify our hypothesis—mmApps can effectively improve patients’ ADE reporting willingness (RR 2.59, 95% CI 1.26-5.30; *P*=.009). This is a brand new result that has not been reported by others. Reporting ADEs and near-miss events was considered an important measure for ensuring medication safety, and information about these events is valuable and can be used to prevent ADEs. The Organization for Economic Co-operation and Development [23] recommends that patients should report ADEs, as ADE reporting does not require enormous financial costs and human resources. Further, ADE reporting is excellent in terms of its value; if done well, ADE reporting can reduce the incidence of harm by 15% and decrease economical burdens, thereby saving millions of dollars each year [24]. mmApps have some merits with regard to increasing ADE reporting willingness. First, they facilitate more communication between physicians and patients [25]. Patients ask physicians for help when they encounter professional terms and confusing problems. Second, patients attach more importance to ADE reporting. Medical staff encourage patients to participate in ADE reporting, and as a result, patients generally realize that their medication safety is considered a top priority. Third, the processing of results is transparent [26]. Patients can use mmApps to obtain feedback about submissions regarding conditions, such as whether a submission is successful and how many people submit the same questions. Additionally, it is convenient for patients to be able to browse through an app to see results as they are processed. We hope to explore more functions that help increase the ADE reporting rate among patients, such as measures for improving patients’ enthusiasm through spiritual encouragement and material reward.

Our study results showed that mmApps can improve medication adherence (RR 1.17, 95% CI 1.04-1.31; *P*=.007). This may be because mmApps reminded patients to take their medicine regularly, provided educational support, and recorded patient histories To some degree, mmApps also strengthened self-effectiveness among patients with chronic diseases and improved quality of life. Degenerative memory, polypharmacy, and comorbidities are common among patients with chronic diseases. As such, they are at high risk of medication nonadherence, and it is not easy to improve medication adherence in this population. Fortunately, we can convey medical solutions through wireless mobile networks, track medications, and even conduct remote consultations with the help of mmApps. Intelligent mmApps empower patients and facilitate self-management at home and abroad. As an auxiliary to physician intervention, mmApps encourage more patients to participate in medical decision-making, thereby improving their disease control capabilities [7]. Further, mmApps are affordable and convenient technologies that rely on existing mobile networks to remotely monitor patients who are difficult to contact or require strict monitoring. Such apps also have the potential to improve the control of risk factors and health conditions. They especially work well for patients with chronic diseases, such as arterial hypertension [27], diabetes [28], and heart failure [29].

Our meta-analysis showed that mmApps had no significant effect on reducing medication errors (RR 0.41, 95% CI 0.13-1.33; *P*=.58). This finding is different from those of other studies. For example, Baumann et al [30] validated a mobile app that was an appropriate and feasible tool for reducing simple calculation and handling errors in drug administration. Moreover, Siebert et al [31] studied a mobile app that reduced prehospital medication errors by providing simulated pediatric resuscitation education. As for our different results, we thought of the following reasons. First, it is possible that patients were not able to identify medication error types well, resulting in the capture of only a small sample medication errors. Second, the studied mmApps may have been limited in terms of their functionality (eg, a lack of accurate identification functions). For instance, an app that scans barcodes on medicine bottle labels is only useful if a patient uses it before taking the medication. Additionally, such apps only provide specific patient and medication information, and they cannot be used to determine whether a medicine bottle has been opened. However, such apps can reduce medication errors by 54% to 87% if a medicine bottle barcode is available and if patients use these apps properly [21]. The third reason is that mmApps have not yet formed a comprehensive and timely information dissemination network and do not fully cover all chronic diseases, resulting in unequal medical information transmission and the easy omission of medication error cases [32]. Although our research showed that mmApps cannot reduce the incidence of medication errors directly, they can be used to achieve medication safety by reminding patients to take their medications regularly and providing health information. Therefore, to some extent, mobile apps can reduce medication errors indirectly. So far, there is no consensus on whether mmApps reduce medication errors. As such, more and higher-level studies are needed to verify their effects on medication errors in the future.

### Limitations

There may be some limitations to this study. First, with respect to the quality assessment, the quality of the included articles was low, and the overall strength of the evidence was moderate. Second, most studies (5/8, 63%) lacked randomization and double-blinding. As such, stricter inclusion criteria and more rigorous randomized controlled trial studies should be considered in the future. Third, the intervention durations were inconsistent. The shortest study was only conducted for 1 month, which could have had an impact on the results. We hoped that the duration of intervention in the included studies would be at least 6 months. Finally, we did not perform a cost-benefit analysis of mmApps, which is important for helping patients decide whether to use an mmApp. Therefore, we plan to do more research on mmApps’ economic and time costs.

### Conclusion

A total of 8 articles were included in this study. We focused on the effects of mmApps on medication safety, and our results showed that mmApps could increase ADE reporting willingness (*P*=.009) and significantly improve medication adherence (*P*=.007) but had little effect on reducing medication errors (*P*=.58). We analyzed several merits of mmApps, with regard to facilitating the willingness to report ADEs; acquired data on how mmApps improved medication adherence among patients with chronic diseases; and found 3 potential reasons for why our medication error results differed from those of other studies.

## Appendix

Multimedia Appendix 1 Search strategy for the literature databases.

## Abbreviations

ADE: adverse drug event
CORA: Chemotherapy Assistant
MeSH: Medical Subject Headings
mmApp: mobile medical app
MMAS: Morisky Medication Adherence Scale
PRISMA: Preferred Reporting Items for Systematic Reviews and Meta-Analyses
RR: relative risk
WHO: World Health Organization

## Data Availability

The data are openly available in a public repository.
