# Supplementary material for: Effectiveness of Mobile Medical Apps in Ensuring Medication Safety Among Patients With Chronic Diseases: Systematic Review and Meta-analysis
Source: JMIR Mhealth Uhealth. 2022 Nov 22;10(11):e39819. doi: 10.2196/39819 (PMC9727690; doi:10.2196/39819)
Supplement: Multimedia Appendix 1 [file mhealth_v10i11e39819_app1.docx]

**Multimedia Appendix 1: Search Strategy**

**Database: Web Of Science<1997 to 2022>**

**Date searched: 31 March 2022**

**Search Strategy:**

**#1 TS=(adverse drug event* OR ADE OR Drug Related Side Effects and Adverse Reaction* OR Drug Side Effects OR Adverse Drug Reaction* OR Side Effects of Drugs OR Drug Toxicity)**

**#2 TS=(medication adherence OR Drug Adherence OR Medication Persistence OR Medication Compliance OR Drug Compliance)**

**#3 TS=(medication errors OR Look-Alike Sound-Alike Medication Errors OR High-Alert Drug Error OR Drug Use Error*)**

**#4 TS=(mobile application*OR mobile App* OR Portable Software App* OR Smartphone Apps OR Portable Electronic Apps OR Portable Electronic Application)**

**#5 #1 OR #2 OR #3**

**#6 #5 AND #4 (256)**

**Database: CINAHL (Cumulative Index to Nursing & Allied Health Literature )<1948to 2022>**

**Date searched: 12 May 2022**

**Search Strategy:**

S1 TX mobile application*OR mobile App* OR Portable Software App* OR Smartphone Apps OR Portable Electronic Apps OR Portable Electronic Application

S2 TX adverse drug event* OR ADE OR Drug Related Side Effects and Adverse Reaction* OR Drug Side Effects OR Adverse Drug Reaction* OR Side Effects of Drugs OR Drug Toxicity

S3 TX medication adherence OR Drug Adherence OR Medication Persistence OR Medication Compliance OR Drug Compliance

S4 TX medication errors OR Look-Alike Sound-Alike Medication Errors OR High-Alert Drug Error OR Drug Use Error*

S5 S2 OR S3 OR S4

S6 S1 AND S5 (21)

**Database: PubMed <2000 to 2022>**

**Date searched: 31 March 2022**

**Search Strategy:**

**(((medication errors[Title/Abstract] OR Look-Alike Sound-Alike Medication Errors[Title/Abstract] OR High-Alert Drug Error[Title/Abstract] OR Drug Use Error*[Title/Abstract]) OR (medication adherence[Title/Abstract] OR Drug Adherence[Title/Abstract] OR Medication Persistence[Title/Abstract] OR Medication Compliance[Title/Abstract] OR Drug Compliance[Title/Abstract])) OR (adverse drug event*[Title/Abstract] OR ADE[Title/Abstract] OR Drug Related Side Effects[Title/Abstract] AND Adverse Reaction*[Title/Abstract] OR Drug Side Effects[Title/Abstract] OR Adverse Drug Reaction*[Title/Abstract] OR Side Effects of Drugs[Title/Abstract] OR Drug Toxicity[Title/Abstract])) AND (mobile application*[Title/Abstract] OR mobile App*[Title/Abstract] OR Portable Software App*OR Smartphone Apps[Title/Abstract] OR Portable Electronic Apps[Title/Abstract] OR Portable Electronic Application[Title/Abstract]**

**Database: CNKI(China National Knowledge Infrastructure) <1999to 2022>**

**Date searched: 10 April 2022**

**Search Strategy:**

**检索条件：**

**[(篇关摘%用药安全 + 服药依从性 + 用药错误 + 药物不良事件 + 安全用药 + 药物管理) AND](https://kns.cnki.net/kns8/AdvSearch?id=118&dbcode=CFLS&searchtype=gradeSearch&ishistory=1" \o "(篇关摘%用药安全 + 服药依从性 + 用药错误 + 药物不良事件 + 安全用药 + 药物管理) AND (篇关摘%移动医疗软件 + 移动医疗APP + 智慧医疗 + 智能手机 + 大数据) AND (篇关摘%老年人 + 慢性病病人 + 慢性病患者 + 高血压 + 糖尿病 + 冠心病)" \t "https://kns.cnki.net/kns8/manage/_blank)**

**[(篇关摘%移动医疗软件 + 移动医疗APP + 智慧医疗 + 智能手机 + 大数据)](https://kns.cnki.net/kns8/AdvSearch?id=118&dbcode=CFLS&searchtype=gradeSearch&ishistory=1" \o "(篇关摘%用药安全 + 服药依从性 + 用药错误 + 药物不良事件 + 安全用药 + 药物管理) AND (篇关摘%移动医疗软件 + 移动医疗APP + 智慧医疗 + 智能手机 + 大数据) AND (篇关摘%老年人 + 慢性病病人 + 慢性病患者 + 高血压 + 糖尿病 + 冠心病)" \t "https://kns.cnki.net/kns8/manage/_blank)**

**[AND](https://kns.cnki.net/kns8/AdvSearch?id=118&dbcode=CFLS&searchtype=gradeSearch&ishistory=1" \o "(篇关摘%用药安全 + 服药依从性 + 用药错误 + 药物不良事件 + 安全用药 + 药物管理) AND (篇关摘%移动医疗软件 + 移动医疗APP + 智慧医疗 + 智能手机 + 大数据) AND (篇关摘%老年人 + 慢性病病人 + 慢性病患者 + 高血压 + 糖尿病 + 冠心病)" \t "https://kns.cnki.net/kns8/manage/_blank)**

**(篇关摘%老年人 + 慢性病病人 + 慢性病患者 + 高血压 + 糖尿病 + 冠心病)**

**检索范围：**

**资源范围:总库；同义词扩展；更新时间:建库至2022年4月10日**

**Database: SinoMed (Chinese biomedical database),<1978to 2022>**

**Date searched: 10 April 2022**

**Search Strategy:**

**#1 主题=（用药安全 OR 服药依从性 OR 用药错误 OR 药物不良事件 OR 安全用药 OR 药物管理）**

**#2 主题=（移动医疗软件 OR 移动医疗APP OR 智慧医疗 OR 智能手机 OR 大数据）**

**#3 主题=（老年人 OR 慢性病病人 OR 慢性病患者 OR 高血压 OR 糖尿病 OR 冠心病）**

**#4 #1 AND #2 AND #3**
